# Three-dimensional aligned nanofibers-hydrogel scaffold for controlled non-viral drug/gene delivery to direct axon regeneration in spinal cord injury treatment

**DOI:** 10.1038/srep42212

**Published:** 2017-02-07

**Authors:** Lan Huong Nguyen, Mingyong Gao, Junquan Lin, Wutian Wu, Jun Wang, Sing Yian Chew

**Affiliations:** 1 School of Chemical and Biomedical Engineering, Nanyang Technological University, Singapore 637459, Singapore; 2 Department of Spine Surgery, Renmin Hospital of Wuhan University, Wuhan 430060, China; 3 School of Biomedical Sciences, The University of Hong Kong Li Ka Shing Faculty of Medicine, Pokfulam, Hong Kong SAR, China; 4 Research Center of Reproduction, Development and Growth, Li Ka Shing Faculty of Medicine, The University of Hong Kong, Pokfulam, Hong Kong SAR, China; 5 State Key Laboratory of Brain and Cognitive Sciences, Li Ka Shing Faculty of Medicine, The University of Hong Kong, Pokfulam, Hong Kong SAR, China; 6 Guangdong-Hongkong-Macau Institute of CNS Regeneration, Jinan University, Guangzhou 510632, PR China; 7 The CAS Key Laboratory of Innate Immunity and Chronic Disease, School of Life Sciences and Medical Center, University of Science & Technology of China, Hefei, Anhui 230027, PR China; 8 Lee Kong Chian School of Medicine, Nanyang Technological University, Singapore 308232, Singapore

## Abstract

Spinal cord injuries (SCI) often lead to persistent neurological dysfunction due to failure in axon regeneration. Unfortunately, currently established treatments, such as direct drug administration, do not effectively treat SCI due to rapid drug clearance from our bodies. Here, we introduce a three-dimensional aligned nanofibers-hydrogel scaffold as a bio-functionalized platform to provide sustained non-viral delivery of proteins and nucleic acid therapeutics (small non-coding RNAs), along with synergistic contact guidance for nerve injury treatment. A hemi-incision model at cervical level 5 in the rat spinal cord was chosen to evaluate the efficacy of this scaffold design. Specifically, aligned axon regeneration was observed as early as one week post-injury. In addition, no excessive inflammatory response and scar tissue formation was triggered. Taken together, our results demonstrate the potential of our scaffold for neural tissue engineering applications.

Spinal cord injuries (SCI) irreversibly disrupt the spinal tracts and ultimately lead to permanent functional impairment. Although the direct administration of biological factors to injury sites is frequently applied, such an approach often does not lead to robust tissue regeneration and reformation due to the rapid biological clearance of these agents from our bodies^1^,^2^,^3^,^4^. Given this limitation, biodegradable scaffolds are increasingly employed as temporary frameworks for sustained delivery of biomolecules and to support neo-tissue formation. To mimic the mechanical properties of the spinal cord, hydrogels and self-assembled peptide nanofibers are commonly used^5^,^6^,^7^,^8^,^9^,^10^. Unfortunately, these scaffolds are often isotropic in architecture and hence lack the ability to direct the growth of regenerated axons through the extensively disorganized injured tissues for proper neuronal reconnections.

We introduce herein a biodegradable, three-dimensional aligned nanofibers-hydrogel scaffold as a biofunctionalized platform to provide contact guidance and sustained non-viral drug/gene delivery for nerve injury treatment. The scaffold comprises of aligned poly (ε-caprolactone-*co*-ethyl ethylene phosphate) (PCLEEP) electrospun nanofibers that were distributed in a three-dimensional configuration within a collagen hydrogel. Comparing to the microchannels and microfibers that were previously used for SCI treatments^11^,^12^,^13^,^14^,^15^, nanofibers more closely imitate the size scale and architecture of the natural extracellular matrix that is present in our body. More importantly, our scaffold design allows robust drug/gene encapsulation. Our previous works have shown that electrospinning enabled the incorporation of a wide variety of drugs ranging from proteins to low molecular weight lipophilic drugs and nucleic acids within nanofiber matrices^16^,^17^,^18^,^19^,^20^,^21^,^22^,^23^,^24^,^25^,^26^. Collagen hydrogel, on the other hand, is well-established in its ability to enable sustained drug/gene delivery^27^,^28^,^29^.

To demonstrate the efficacy of our PCLEEP-collagen hybrid scaffold in achieving effective local drug/gene delivery *in vivo*, we incorporated neurotrophin-3 (NT-3) as the model protein and miR-222 as the model microRNA. NT-3 is known to promote neuronal survival, axonal sprouting and regeneration^30^,^31^,^32^,^33^. In addition, it facilitates the proliferation and differentiation of oligodendrocyte precursors cells^30^,^34^,^35^. On the other hand, miR-222 is enriched in axons and participates in controlling local protein synthesis at distal axons^36^,^37^,^38^. Such controlled local protein synthesis plays crticial roles in allowing severed axons to undergo regeneration within hours after injuries, independent of protein transport from the cell soma of neurons^36^,^39^. Unfortunately, the expression of miR-222 is often significantly altered after nerve injuries^40^,^41^,^42^,^43^,^44^,^45^,^46^. *In vitro,* when miR-222 was deliberately over-expressed in injured adult neurons, enhanced regrowth was observed^40^,^41^,^47^,^48^. However, the use of miR-222 to enhance nerve regeneration after SCI has not been attempted. We speculate that this is largely due to the lack of effective non-viral methods to deliver microRNAs to neurons *in vivo*. Thus, in this study, we incorporated NT-3 and miR-222 into the PCLEEP-collagen hybrid scaffold to evaluate the feasibility of a single scaffold in imparting synergistic biochemical and topographical signals to enhance nerve regeneration after SCI. The results showed that our biofunctionalized scaffolding platform effectively provided bio-mimicking contact guidance, and allowed the controlled delivery of various drugs and therapeutic biomolecules of drastically different nature and molecular weights.

## Materials and Methods

### Scaffold fabrication

#### Preparation of micellar nanoparticles

The micellar nanoparticles (MNP), which consist of poly(ε-caprolactone)-block-polyethylene glycol (PCL-PEG) and poly(ε-caprolactone)-block-poly(2-aminoethyl ethylene phosphate) (PCL-PPEEA), were prepared according to the literature^49^. Briefly, 10 mg of PCL_4.8k_-PEG_3.4k_ and PCL_29_-PPEEA_21_ (molar ratio = 1.5/1) were dissolved in 500 μl of solvent mixture, which comprised of methanol and acetonitrile (volume ratio = 1/1). Ultrapure water (5.0 ml) was added drop by drop into the polymer solution during stirring. After stirring at room temperature for 2 h, the chemical solvent was removed by vacuum. The MNP concentration was then adjusted to 2 mg/mL by adding extra ultrapure water. This MNP solution was kept at 4 °C until used.

#### Aligned PCLEEP fibers

The PCLEEP copolymer (Mw = 59,102, Mn = 25,542) was synthesized by previously reported methods^43^,^50^. To fabricate aligned PCLEEP nanofibers, 35 wt% of PCLEEP was dissolved in 2,2,2-Trifluoroethanol (TFE, Sigma-Aldrich) and electrospun using the two-pole air gap electrospinning technique^51^. Specifically, the spinning solution was loaded into a 1 ml syringe that was capped with a 22 gauge blunt-tipped needle. This solution was then charged at +10 kV and released at a flow rate of 1.9 ml/h. The PCLEEP fibers were then deposited within a 5.0 cm air gap area that was between two stationary collector poles (−4.0 kV). Each set of fibers was obtained after 90 seconds of spinning. These fiber bundles were then sterilized under UV light for 30 minutes.

To fabricate PCLEEP nanofibers that encapsulated nucleic acids, 40 wt% of PCLEEP in TFE was used. First, Cy5-labeled oligonucleotides (ODN), 488-ODN, NEG-miR (100 μM) or miR-222 was complexed with MNP (2.0 mg/ml) at room temperature for 10 minutes. Thereafter, the nucleic acid complexes were added into the PCLEEP solution at a RNA/MNP/TFE ratio of 35 μl/296 μl/1 ml. Following that, this polymer solution was dispensed at a flow rate of 0.5 ml/h and electrospun at an electrical voltage of 13 kV. Each set of fibers was obtained after 60 seconds of electrospinning.

#### Scaffolds

Rat-tail Collagen type I (10.08 mg/ml, Corning, LOT 4041009) was used to fabricate hydrogel according to the manufacturer’s protocol (BD Biosciences). Briefly, 10x phosphate buffered saline (PBS; pH 7.4, Life Technologies), collagen type I, 1.0 N NaOH, and de-ionized (DI) water were added into a sterile microtube and mixed properly to get a final collagen concentration of 6.0 mg/ml. The collagen mixture was then kept on ice until used. For neurotrophin-3 (NT-3)-incorporation, 0.5 μl of 10x PBS and 4.5 μl of DI water in the collagen mixture were substituted by 5 μl of 0.1% bovine serum albumin (BSA, Sigma-Aldrich) containing human NT-3 (4.0 μg/μl, PeproTech) and heparin (400 μg/μl) (1:1 v/v). The theoretical loading of NT-3 was 2 μg/animal. For Cy5-ODN, 488-ODN, or miRNA-incorporation within collagen matrices, 2.6 μl of Cy5-ODN, 488-ODN, or miRNA (100 μM) were complexed with 22 μl of MNP (2.0 mg/ml) for 10 minutes at room temperature. Following that, the miRNA complex was added into the collagen mixture in place of 24.6 μl of DI water. The theoretical loading of miRNA was 0.76 μg/animal. To obtain scaffolds with aligned nanofibers, a sterilized cylinder mold (5.0 mm in length and 4.5 mm in inner diameter) was pre-set with 4 sets of fibers in the core region prior to the addition of the collagen mixture. Hydrogel formation took place at 37 °C in the incubator for 45 minutes. Thereafter, the scaffolds were frozen at −80 °C then lyophilized and kept at −20 °C until used.

### Scaffold characterization

#### Fiber diameter

The nanofibers and the scaffolds were sputter-coated with platinum (JEOL, JFC-1600) at 10 mA for 120 s and 140 s, respectively. Thereafter, the samples were observed under the scanning electron microscope (SEM; JEOL, JSM-6390 LA) at an accelerating voltage of 10 kV. The fiber diameters were measured using ImageJ software (http://imagej.nih.gov/ij/) on the SEM images. At least 100 fibers were measured.

#### Scaffold degradation

Each scaffold (5.0 mm in length, 1.8 ± 0.11 mg) was immersed in 1.2 ml of PBS in a microtube and incubated at 37 °C. To quantify the total scaffold degradation rate, five samples were used. At each designated time point, each scaffold was retrieved, slightly taped against the inner wall of the microtube for 10 s to remove excess water. Thereafter, its weight was recorded. The percentage mass loss was then determined as the ratio of the change in weight to the original weight of the scaffold. Results are presented as mean ± standard deviation (S.D.).

#### NT-3 release kinetic

The release kinetics of NT-3 from the scaffold was evaluated under static conditions. Briefly, three NT-3 encapsulated scaffolds (2.14 ± 0.15 mg) were immersed in PBS (1.2 ml of PBS per scaffold) and incubated at 37 °C. At each time point, 600 μl of PBS was collected and replaced by an equal volume of fresh PBS. The supernatant was then used to measure the amount of NT-3 that was released from the scaffolds over time using the ELISA assay (RnD Systems), following manufacturer’s protocol. After 3 months, the scaffolds were retrieved and fully dissolved in 300 μl of collagenase type 1 (Life Technologies) at 37 °C for 30 minutes. Thereafter, the supernatant was used to measure the remaining amount of NT-3 within the scaffolds by ELISA assay. Thus, the total amount of NT-3 retrieved from the scaffold was the NT-3 cumulative release amount plus the remaining amount of NT-3 retrieved after dissolving the scaffold. The experimental loading efficiency of NT-3 was then computed using the following equation:


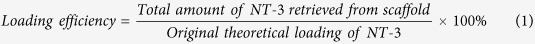


The cumulative release profile of NT-3 (%), after taking into account the loading efficiency, is finally presented as mean ± standard deviation (S.D.).

#### microRNA release kinetics

The experimental loading efficiency of microRNA within the collagen hydrogel was evaluated after lyophilization. Briefly, NEG-miR-incorporated scaffolds (n = 3) were fully dissolved in 300 μl of collagenase type 1 at 37^ ^°C for 30 minutes. The amount of NEG-miR within the extracted solution was determined by Quant-iT^TM^ RiboGreen® RNA reagent kit (Invitrogen) after de-complexion from MNP using heparin (Sigma-Aldrich, 20 μg/ml). The experimental loading efficiency of miR was computed using the following equation:


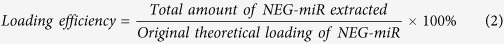


The release kinetics of miR from the scaffold was evaluated under static conditions. NEG-miR-incorporated scaffolds (1.55 ± 0.1 mg) were incubated in 1.2 ml 1x Tris-EDTA buffer (TE buffer; pH 7.4, 1st BASE) at 37 °C. Three samples were used for each time point during this study. At each time point, 600 μl of TE buffer was withdrawn and replaced by an equal volume of fresh buffer. The supernatant was then used to measure the amount of miR that was released over time using Quant-iT^TM^ RiboGreen® RNA reagent kit after treating with heparin (20 μg/ml). The cumulative release of miRNA (%), after taking into account the loading efficiency, is presented as mean ± standard deviation (S.D.).

#### Spinal cord injury and scaffold implantation

Adult female Sprague Dawley rats (6–8 weeks, 200–250 g) were obtained from In Vivos Pte Ltd (Singapore). All experimental procedures were approved by the Institutional Animal Care and Use Committee, Nanyang Technological University (IACUC, NTU). All methods were performed in accordance with the relevant guidelines and regulations.

Rats were anesthetized using a combination of ketamine (73 mg/kg) and xylazine (7.3 mg/kg) right before surgery. The surgical field was shaved and cleaned with 70% ethanol and then Betadine. Thereafter, the dorsal skin was incised 3–4 cm caudally from the base of the head. Muscle layers were opened along the midline to gain access to the cervical spine. Following that, the C5 spine was located using T2 process as an anatomic landmark and its right side was opened to expose the C5 spinal cord. Afterward, the dura was cut open and a 1/3 incision was made using a pair of fine micro-scissors. Thereafter, scaffolds (1.0 mm in length) were implanted into the incision site such that the nanofibers were aligned to the longitudinal axis of the spinal cord. Subsequently, the dura was sutured back and a layer of fat tissue was used to cover the injury area. The muscle was then sutured and the skin was closed with auto clips. Animals were randomly divided into five treatment groups as presented in Table 1.

All animals were injected with buprenorphine subcutaneously (0.05 mg/kg) twice a day for 3 days post-operation. Subsequently, they were fed with meloxicam (5.0 mg/ml, 1.0 ml in 500 ml drinking water) continuously for the following 7 days.

#### Immunohistochemistry

At 7, 10, 14, and 28 days post-injury, animals were perfused with 0.9% saline followed by 4% ice-cold paraformaldehyde (Millipore). After perfusion, 5 cm of spine containing the spinal cord with the lesion site was harvested from each animal and post-fixed for 16–18 h at 4 °C before transferring to 15% sucrose for 24 h. Thereafter, 1.5 cm of the spinal cords containing the lesion sites were removed from the spines and stored in 30% sucrose at 4 °C until cryosectioned. Spinal cord samples were sectioned coronally on a cryostat set at 25 μm thickness. Subsequently, the spinal cord sections were blocked in 5% normal goat serum (NGS, Sigma-Aldrich) and 0.1% Triton X-100 (Sigma-Aldrich) for 1 h at room temperature. The sections were then incubated with primary antibodies diluted in the above block solution at 4 ^o^C overnight followed by secondary antibodies diluted in PBS for 2.5 h at room temperature. The following primary antibodies were used respectively: rabbit anti-neurofilament 200 (NF, Sigma-Aldrich, 1:700 dilution), chicken anti-NF (Biolegend, 1:1000), rabbit anti-glial fibrillary acidic protein (GFAP, clone GA5, Millipore, 1:1000 dilution), mouse anti-myelin associated glycoprotein (MAG, clone 513, Millipore, 1:200 dilution), and mouse anti-CD11b/c (anti-Ox42, Abcam, 1:200 dilution). Secondary antibodies were Alexa Fluor 488-conjugated Goat Anti-Mouse/Rabbit IgG (Invitrogen, 1:700 dilution) and Alexa Fluor 555-conjugated Goat Anti-Mouse/Rabbit IgG (Invitrogen, 1:1000 dilution). Nuclear staining was performed by incubating the sections with DAPI (1:1000, Life Technologies) at room temperature for 10 minutes after the secondary antibodies. Thereafter, all samples were examined using an Olympus IX71 inverted microscope and a Zeiss LSM710 META confocal microscope.

### Statistical analysis

All data are presented as mean ± standard deviation (S.D.). Statistical comparison for fiber diameter, after verifying to have equal variances, was analyzed using one-way ANOVA followed by Turkey post-hoc test.

## Results and Discussion

Many growth inhibitory factors exist after SCI. Among these, the formation of a cyst, which is a fluid filled cavity, has been a long withstanding problem. In the absence of an underlying supporting substrate, nerve/tissue regeneration is poor. As such, tissue scaffolds have been explored to provide bridges for nerve regrowth across injuries. Among the scaffolds analyzed, hydrogels^8^,^14^,^52^,^53^,^54^,^55^,^56^,^57^,^58^ and self-assembled peptide nanofibers^59^,^60^ are most popular. However, the directions in which nerve regenerates within these scaffolds are always haphazard, often resulting in very few axons exiting the implant and reconnecting with host tissues. Here, we hypothesize that biomimicking aligned nanofiber topography is beneficial in guiding axonal regeneration. In addition, nerve regeneration may be further assisted by the synergistic supplementation of biomoleucles such as neurotrophic factors (e.g. NT-3) and microRNAs (e.g. miR-222).

While the role of neurotrophic factors in nerve regeneration is well-recognized, the involvement of microRNAs and RNA interference (RNAi) are only beginning to be elucidated. RNAi is widely adopted in treatments related to cancer therapy and genetic diseases. However, RNAi is also useful in regenerative medicine. Unfortunately, one of the major stumbling blocks to the translational use of RNAi is the development of proper non-viral delivery systems for effective and efficient delivery with minimal off-target effects in the body. Traditional approaches of systemic delivery for cancer and genetic disease therapy does not benefit traumatic tissue injuries since the latter often involves confined injuries that require the reestablishment of tissue architecture. MiRNA delivery using scaffolds, as demonstrated here, represents a more appropriate approach since scaffolds provide the necessary structural support and directional cues for tissue regrowth and restructuring. Scaffolds also provide localized delivery of drugs, hence minimizing off-target systemic side effects. In terms of neuronal regrowth, controlling the intrinsic growth ability of neurons by miRNAs will provide more robust treatment targets and outcomes. This is because although growth factor therapy is commonly used, most factors offer selective support for only the subclass of neurons that express the appropriate receptors to allow neurons to take up these factors. In cases where the expression levels of the receptors are low and nerve damage involves a wide spectrum of neurons (such as in diabetes), growth factor-based approaches are ineffective^61^.

**Figure 1:**
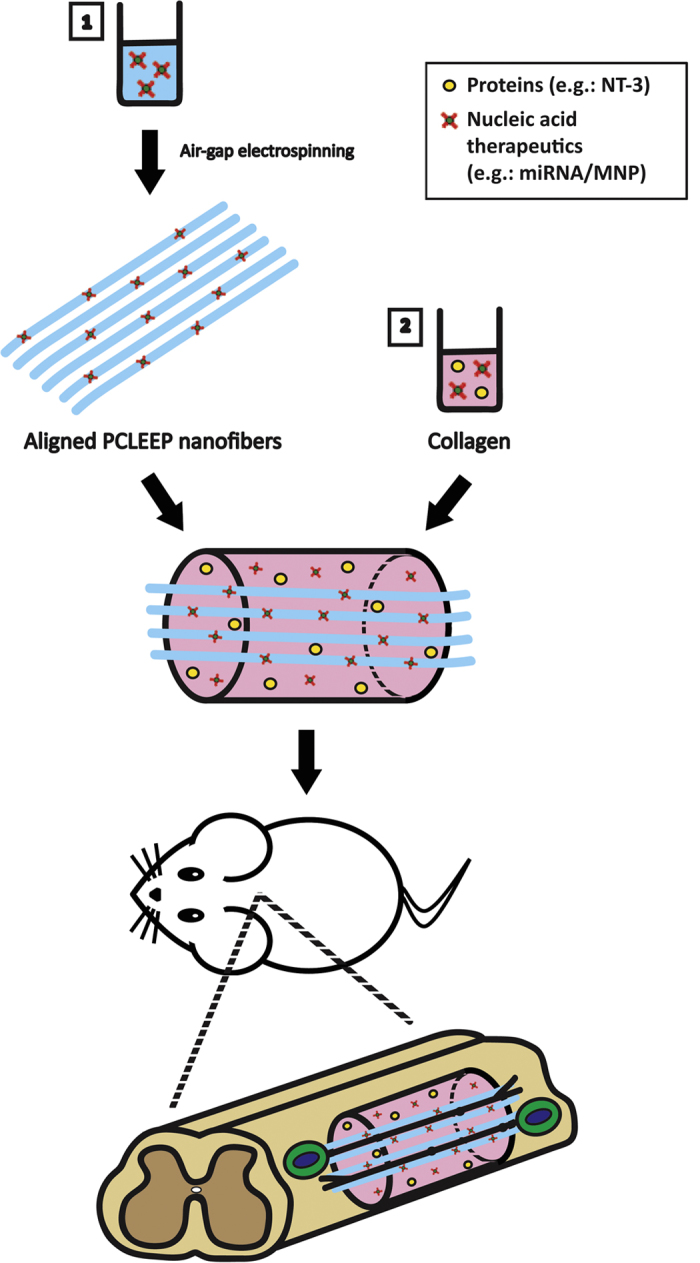
Schematic illustration of nanofibers-hydrogel scaffold fabrication process and implantation into the spinal cord. Drugs may be loaded into the scaffolds by direct encapsulation (Step 1) into electrospun nanofibers or (Step 2) within collagen hydrogel.

**Figure 2:**
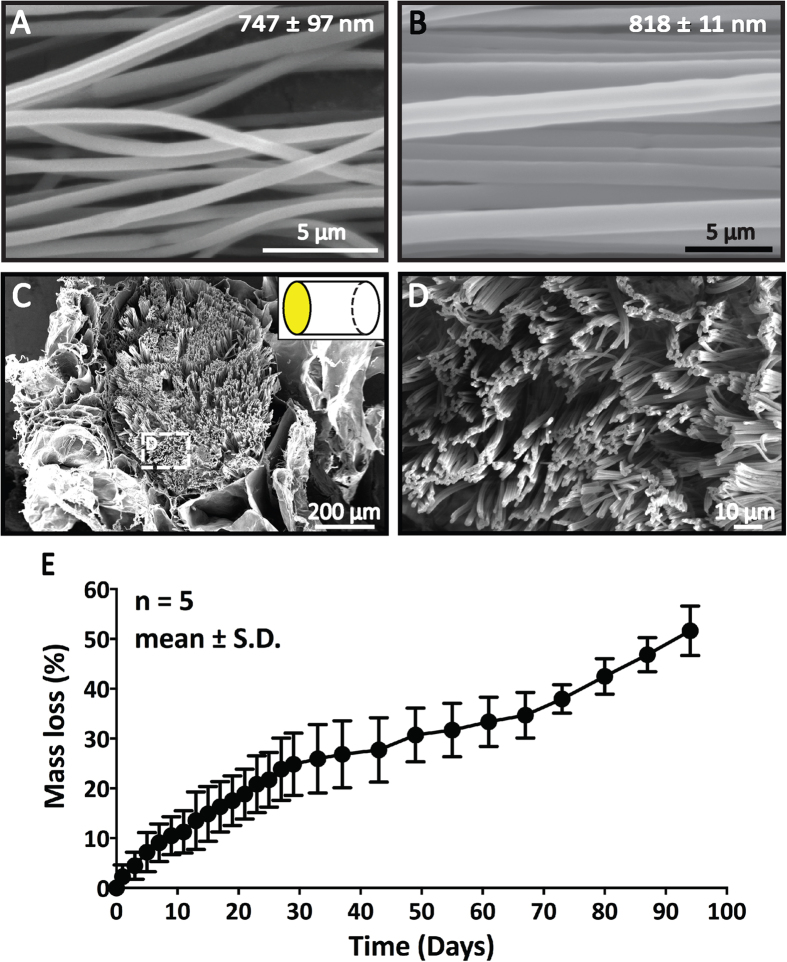
Scaffold characterization. (**A-D**) SEM images of scaffolds. (**A,B**) Aligned PCLEEP nanofibers (**A**) with and (**B**) without encapsulation of microRNA (NEG-miR). (**C,D**) Cross-sectional view of nanofibers-hydrogel scaffold. (**D**) High magnification image of inset in (**C**). (**E**) *In vitro* degradation rate of the nanofibers-hydrogel scaffold.

MiRNAs are labile molecules. As such, the incorporation of miRNAs into PCLEEP-collagen scaffolds was an attempt to protect these drugs from biodegradation (Fig. 1). Here, we demonstrate the encapsulation of miRNAs by electrospinning to achieve non-viral delivery of these nucleic acid therapeutics. In our scaffold, miRNA incorporation did not alter the morphology and alignment of the electrospun fibers. Specifically, bead-free and uniformly aligned PCLEEP nanofibers were fabricated with and without miRNA encapsulation (Fig. 2A and B). Their average fiber diameters were 747 ± 97 nm and 818 ± 11 nm respectively and were not significantly different.

**Figure 3:**
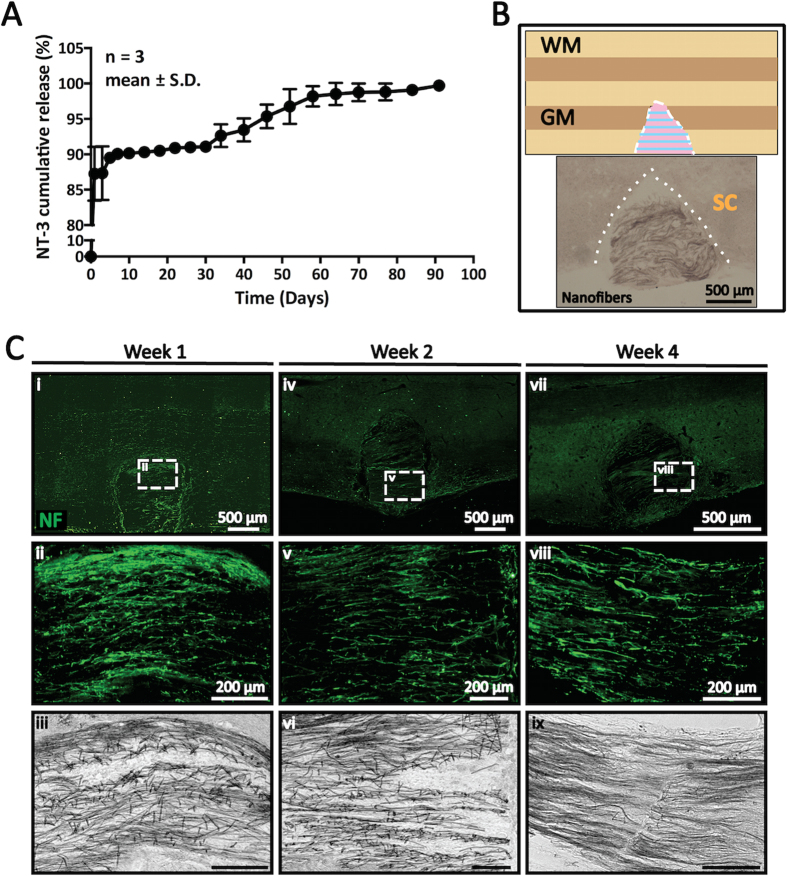
*In vivo* regeneration of aligned neurofilaments (NF^+^, green) within NT-3-incorporated nanofibers-hydrogel scaffolds after spinal cord injury. (**A**) *In vitro* NT-3 release kinetics of NT-3-incorporated nanofibers-hydrogel scaffolds. (**B**) Schematic illustration and corresponding light micrograph depicting nanofibers-hydrogel scaffolds that were implanted into spinal cord tissues. Dotted line: tissue-implant interface. SC: spinal cord tissue. (**C**) *In vivo* regeneration of aligned neurofilaments within injury site at (i–iii) 1 week, (iv–vi) 2 weeks and (vii–ix) 4 weeks post-injury. (ii, v, and viii) High magnification images of insets in i, iv, and vii respectively. (iii, vi, and ix) Corresponding bright-field images of nanofibers implanted within spinal cord tissues for (ii, v, and viii) respectively.

**Figure 4:**
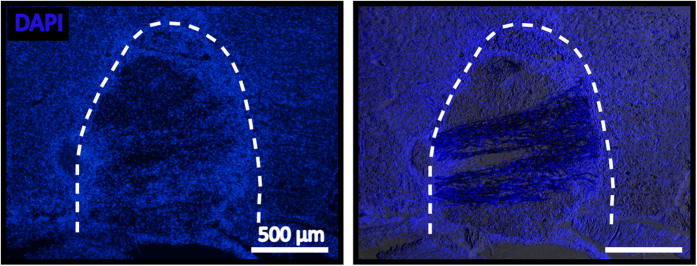
Extensive cellular infiltration into nanofibers hydrogel scaffolds at 1 week post implantation. Left: DAPI staining for cell nuclei. Right: Merged image of DAPI staining and bright field showing scaffold with aligned nanofibers. Dotted line: implant-tissue interface.

**Figure 5:**
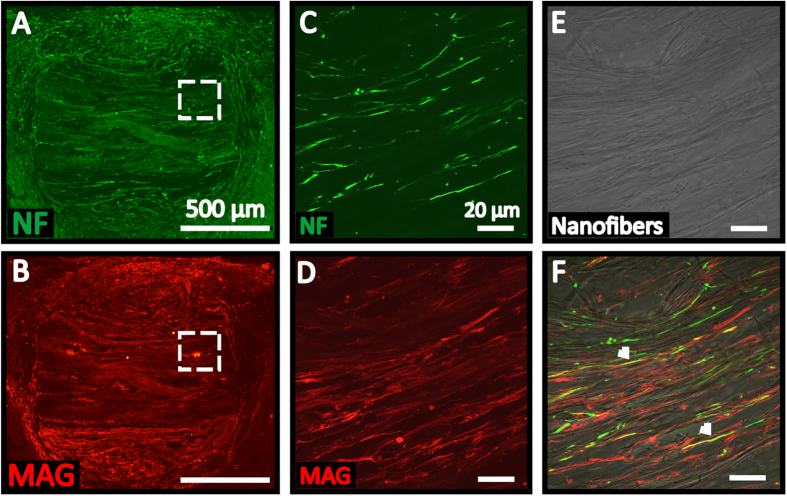
*In vivo* regeneration of aligned neurofilaments (NF^+^, green) and remyelination (MAG^+^, red), which colocalized within the nanofibers-hydrogel scaffolds at 4 weeks post-implantation. (**A,B**) Overview of the representative longitudinal spinal cord section. (**C,D**) High magnification images of the insets in (**A** and **B**) respectively. (**E**) Corresponding bright-field image of nanofibers in (**C** and **D**). (**F**) Merged images of (**C**,**D**, and **E**). Arrow heads indicate colocalization of NF-MAG signals.

**Figure 6:**
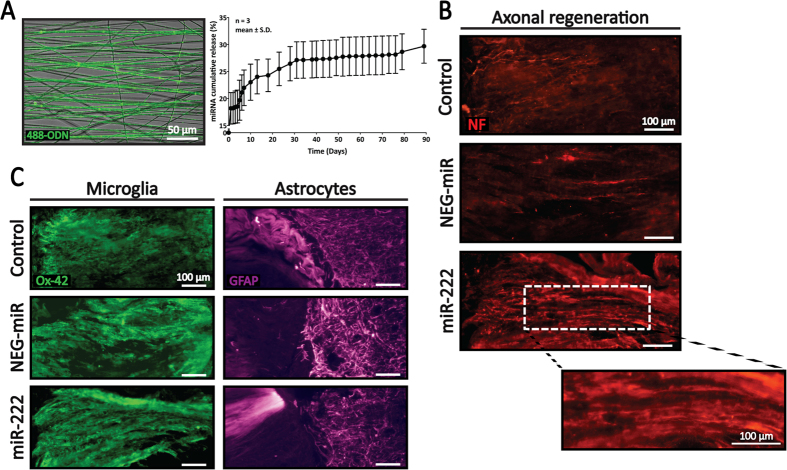
Enhanced regeneration of aligned neurofilaments (NF^+^, red) within nanofibers-hydrogel scaffolds that incorporated miR-222 at 10 days post-implantation. (**A**) Distribution of 488-ODN that was encapsulated within PCLEEP electrospun fibers and microRNA release profile of the nanofibers-hydrogel scaffold. (**B**) Neurofilament regeneration within nanofibers-hydrogel scaffolds at 10 days post-implantation. (**C**) Evaluation of microglia (Ox42^+^, green) and astrocyte (GFAP^+^, magenta) reactions within nanofibers-hydrogel scaffolds depicted no adverse side effects in the presence of miR-222.

Besides serving as a drug/gene delivery scaffold, our PCLEEP-collagen hybrid substrate provides aligned topographical signals for synergistic contact guidance effect over neuronal regeneration. As compared to hydrogels and micron-sized structures, nanofibers more closely imitate the topographical features of the natural extracellular matrix. By combining aligned electrospun nanofibers with collagen hydrogel, the orientation and alignment of PCLEEP nanofibers were retained even after implantation into the injured spinal cord (Fig. 3). Close examination of the nanofibers-hydrogel scaffold revealed the presence of loosely packed, three-dimensionally distributed aligned nanofibers within the collagen gel (Fig. 2C and D). This loose arrangement of aligned fibers in turn facilitated robust *in vivo* cell penetration (Fig. 4) and neurite infiltration (Figs 3, 5, and 6).

Aside from providing a biomimicking 3D architecture, collagen possesses inherent cell adhesivity that supports cell attachment as compared to other biomaterials that have been widely explored for SCI treatment, such as agarose, chitosan, and fibrin^4^. To evaluate the stability and degradability of the scaffold, we examined its *in vitro* degradation rate under physiologically-relevant conditions. As shown in Fig. 2E, the scaffolds were gradually degraded overtime. In particular, total mass losses of ~24.8, 33.4, and 51.6% were respectively reported after 1, 2, and 3 months *in vitro*. In addition, our previous *in vivo* study showed that PCLEEP fibers retained their morphology and alignment after 3 months post-implantation into the rat’s spinal cords^33^. Thus, degradation occurred mostly within the collagen hydrogel throughout the duration of our studies.

NT-3 is a neurotrophic factor commonly used for SCI treatment to promote neuronal survival and axonal regrowth^30^,^31^,^32^,^33^. Here, we incorporated NT-3 into the collagen hydrogel matrix for localized and controlled delivery. In order to regulate the release rate of NT-3, heparin was added in combination with NT-3^23^. A total loading of 54.6 ng NT-3/mg scaffold was achieved with a loading efficiency of 1.17%. As indicated in Fig. 3A, ~90.1% of NT-3 was released within the first week. Thereafter, the release rate of NT-3 was decreased and steady for up to 3 months (99.7 ± 0.07%). This release profile likely resulted from the non-specific binding of NT-3/heparin to the collagen hydrogel. Nonetheless, the initial supplementation of NT-3 after SCI is essential to minimize neuronal apoptosis as well as support neurite regrowth^7^. Correspondingly, we observed robust axonal regeneration at 1, 2, and 4 weeks post-implantation (Fig. 3C). Furthermore, when compared to currently available scaffold systems, our PCLEEP-collagen hybrid scaffold prolonged the availability of NT-3. For example, using the same platform of NT-3/heparin nonspecific binding with collagen, our previous electrospun nanofiber scaffold almost completely released NT-3 (99.2 ± 3.91%) after only 2 months versus 3 months as seen in our current platform^23^. Furthermore, as compared to our current scaffold, a notably lower amount of NT-3 could be loaded into the nanofiber construct (~21 ng NT-3/mg scaffold^23^). In another example, biodegradable poly(ethylene glycol) hydrogels fully released NT-3 over a 2-week period albeit possessing a lower burst release after 24 h (40% vs 87% in our system)^62^. Similarly, the fibrin scaffold by Taylor *et al*. could only deliver NT-3 for 2 weeks post-implantation due to the rapid scaffold degradation rate^63^. This rapid scaffold degradation further resulted in the formation of cystic cavities at 12 weeks after injury. Meanwhile, our PCLEEP-collagen hybrid scaffold integrated well with the host tissues and showed no sign of cyst formation (Figs 3C and 4) throughout this study and even after 12 weeks post-implantation^33^.

The effectiveness of our NT-3 incorporated scaffolds in supporting axonal ingrowth is depicted in Fig. 3. Besides providing good host-implant integration, aligned regenerated axons were found extensively throughout the implant as early as one week post-implantation. The extent of regeneration was sustained even at later time points of 2 (Fig. 3Civ and v) and 4 weeks post-implantation (Fig. 3Cvii and viii). As compared to our previous work^33^, in this study, we deliberately maximized the loading capacity of our scaffold with twice the amount of NT-3. Correspondingly, the degree of axonal regrowth was also significantly enhanced. Notably, the newly regenerated axons were found to penetrate the scaffolds, following nanofiber alignment (Fig. 3C). These results clearly indicate the contact guidance role of our scaffold in directing *in vivo* axon regeneration.

We next extended our analysis to evaluate the efficacy of the nanofibers-hydrogel scaffolds in supporting *in vivo* remyelination. As shown in Fig. 5, immunostaining for myelin-associated glycoprotein (MAG, a marker of oligodendrocyte myelination) revealed that MAG^ + ^structures were extensively observed throughout the scaffolds at 4 weeks post-implantation (Fig. 5B) and co-localized with NF^ + ^axons (Fig. 5C, D, and F). This result suggests the ability of our PCLEEP-collagen hybrid scaffold in facilitating axonal remyelination *in vivo*. In addition, these MAG^ + ^structures were extended along the aligned PCLEEP nanofibers (Fig. 5C, D, and F), further highlighting the contact guidance effect of our substrates.

Although our scaffold implantation resulted in the extensive axonal ingrowth and remyelination, this 1/3 incision SCI model would not be sufficiently severe to result in significant differences in rat locomotion^33^. We believe that the spontaneous recovery process following incomplete SCIs contributed to this outcome^33^,^64^,^65^,^66^,^67^. Thus, in this study, behavior recovery was not assessed. Future works would focus on using the complete transection model to evaluate functional outcomes.

Apart from protein encapsulation, our scaffolds also allowed the easy incorporation of nucleic acids for non-viral gene transfection. MiRNAs can be encapsulated either into nanofibers during the electrospinning process and/or into the collagen hydrogel (Fig. 1). Notably, the encapsulated oligonucleotides were distributed uniformly along the electrospun nanofibers and did not alter nanofiber architecture (Fig. 6A). By incorporating miRNAs into both nanofibers and collagen hydrogel, a total loading of 0.5 μg microRNA/mg scaffold was achieved with an experimental loading efficiency of ~16%. This loading efficiency is found to be much higher as compared to NT-3. We speculate this greater retention of miRNA inside the scaffold to be due to charge interaction and molecular size. Since miRNA/MNP complexes are slightly positive and small in size, they can be homogenously entrapped inside the neutralized collagen hydrogel with a net charge of ~0^68^. In contrast, heparin is highly negatively charged and large in size, making it hard to be retained inside the collagen hydrogel. As shown in Fig. 6A, a total of 27.1 ± 3.38% miRNA was rapidly released within the first month. Thereafter, the miRNA release rate was decreased and steady for at least another 2 months. This release kinetic of small, non-coding RNAs from our PCLEEP-collagen hybrid scaffold was greatly improved as compared to other existing delivery systems. In particular, Krebs *et al*. demonstrated the release of short-interfering RNA (siRNA) for only ~2 weeks from collagen hydrogels^69^. The mechanism of siRNA/miRNA release over time is likely a combination of polymer charge, hydrogel porosity and biodegradation. It is possible that our high collagen concentration hydrogel (6.0 mg/ml) might have favored RNA retention as compared to Krebs’ 3.0 mg/ml collagen hydrogel.

To evaluate the capacity of our scaffold in providing localized gene delivery *in vivo,* scaffolds incorporated with Cy5-labeled double stranded oligonucleotides (Cy5-ODN, which were of similar size as microRNAs) were implanted into the same SCI model. After 14 days post-implantation, Cy5-ODN was detected in the surrounding tissues up to a distance of ~300 μm from the edge of the scaffolds (Supplementary Figure 1). This finding suggests the ability of our PCLEEP-collagen hydrogel scaffold in providing localized and sustained delivery of miRNAs.

Within the body, axons can extend for long distances. For efficient cell function, protein synthesis occurs locally at the terminal and growth ends of axons so that the transport of biomolecules from the cell soma is not critical. Such controlled local protein synthesis at distal axons has allowed severed axons to undergo regeneration within hours after injuries^35^,^45^. This local protein synthesis is finely controlled by microRNAs, such as miR-222. Indeed, miR-222 enhanced axonal regrowth in injured adult neurons *in vitro*^40^,^41^,^47^,^48^. However, there is no study on miR-222′s effects on SCI treatment to date. Given this knowledge gap, we next closely examined the efficacy of scaffold-mediated miR-222 delivery in supporting *in vivo* axon regeneration. As shown in Fig. 6B, after 10 days post-implantation, robust neurite ingrowth was observed in PCLEEP-collagen hybrid scaffolds that incorporated miR-222. This axon regeneration was more extensive as compared to the plain scaffolds (Control) and scaffolds that encapsulated NEG-miR. Moreover, the incorporation of microRNAs did not alter the integration of the nanofibers-hydrogel implants with surrounding host tissues (Fig. 6C). In particular, no significant difference was observed in microglia and astrocyte reactions among the three experimental groups. Taken together, these results demonstrate the efficacy of our scaffolds in providing effective non-viral delivery of bioactive miR-222 to promote nerve regeneration after SCI.

The process of tissue repair is contributed by a complex cascade of signaling pathways involving various biological factors^70^,^71^,^72^. Hence, spatially controlled and sequential release of drugs could enhance the tissue regeneration process^70^,^73^. Accordingly, the flexibility of our scaffold design allows multiple drugs to be incorporated in different locations of the scaffold, either in the nanofibers or the hydrogel. The different nature of nanofibers and hydrogel would allow drugs to be released with distinct release kinetics, possibly mimicking the natural healing process. Such combinational delivery of drugs, such as miRNAs with growth factors, in the scaffold may be exploited for SCI treatment. For example, after SCI, a dramatic loss of oligodendrocytes (OLs) occurs, which frequently results in axonal demyelination^74^. To replenish this OL pool, oligodendrocyte precursor cells (OPCs) proliferate and are recruited to the lesion site for differentiation and remyelination^74^,^75^. Many growth factors have been known to promote OPC proliferation, such as PDGF-A, FGF-2, and IGF^74^. On the other hand, miR-219/338 enhance OPC differentiation and maturation^75^. Thus, a possible strategy may be to combine these growth factors and miR-219/−338 in our scaffold platform for future SCI treatments.

## Conclusions

In this study, we introduced an aligned nanofibers-hydrogel scaffold as a promising bio-functional platform for nerve injury treatment. Our scaffold provided localized and sustained release of drugs and nucleic acid molecules for non-viral transfection to enhance axon regeneration and remyelination *in vivo*. Since non-viral drug/gene delivery was employed, the application of our scaffolds may be translated to the clinical setting without raising biosafety concerns associated with viral-mediated methods. Additionally, the aligned nanofibers provided topographical signals that effectively directed neurite extensions and supported remyelination within the lesion sites. These scaffolds also demonstrated good host-implant integration. Further works are currently ongoing to evaluate in detail, the ability of our nanofibers-hydrogel scaffold in supporting functional neuronal reconnections, remyelination, and functional recovery after SCI.

## Additional Information

**How to cite this article:** Nguyen, L. H. *et al*. Three-dimensional aligned nanofibers-hydrogel scaffold for controlled non-viral drug/gene delivery to direct axon regeneration in spinal cord injury treatment. *Sci. Rep.* **7**, 42212; doi: 10.1038/srep42212 (2017).

**Publisher's note:** Springer Nature remains neutral with regard to jurisdictional claims in published maps and institutional affiliations.

## Supplementary Material

Supplementary Information

## Figures and Tables

**Table 1:** Experimental groups.

	Group	Description	Number of animals
1	Control	Animals received plain scaffolds	N = 3
2	Cy5-ODN	Animals received cy5-ODN encapsulated scaffolds	N = 4
3	NEG-miR	Animals received NEG-miR encapsulated scaffolds	N = 3
4	miR-222	Animals received miR-222 encapsulated scaffolds	N = 3
5	NT-3	Animals received NT-3 encapsulated scaffolds	N = 11

## References

[b1] Pardridge W. M. Drug transport across the blood-brain barrier. Journal of cerebral blood flow and metabolism: official journal of the International Society of Cerebral Blood Flow and Metabolism 32, 1959–1972, doi: 10.1038/jcbfm.2012.126 (2012). PMC3494002 22929442

[b2] Tester N. J., Plaas A. H. & Howland D. R. Effect of body temperature on chondroitinase ABC’s ability to cleave chondroitin sulfate glycosaminoglycans. Journal of neuroscience research 85, 1110–1118, doi: 10.1002/jnr.21199 (2007). 17265470

[b3] Tsintou M., Dalamagkas K. & Seifalian A. M. Advances in regenerative therapies for spinal cord injury: a biomaterials approach. Neural Regeneration Research 10, 726–742, doi: 10.4103/1673-5374.156966 (2015). 26109946 PMC4468763

[b4] Straley K. S., Po Foo C. W. & Heilshorn S. C. Biomaterial Design Strategies for the Treatment of Spinal Cord Injuries. Journal of Neurotrauma 27, 1–19, doi: 10.1089/neu.2009.0948 (2010). 19698073 PMC2924783

[b5] Drury J. L. & Mooney D. J. Hydrogels for tissue engineering: scaffold design variables and applications. Biomaterials 24, 4337–4351 (2003). 12922147 10.1016/s0142-9612(03)00340-5

[b6] Slaughter B. V., Khurshid S. S., Fisher O. Z., Khademhosseini A. & Peppas N. A. Hydrogels in regenerative medicine. Advanced materials (Deerfield Beach, Fla.) 21, 3307–3329, doi: 10.1002/adma.200802106 (2009). PMC4494665 20882499

[b7] Taylor S. J., McDonald J. W. 3rd & Sakiyama-Elbert S. E. Controlled release of neurotrophin-3 from fibrin gels for spinal cord injury. Journal of controlled release: official journal of the Controlled Release Society 98, 281–294, doi: 10.1016/j.jconrel.2004.05.003 (2004). 15262419

[b8] Wei Y.-T. . Hyaluronic acid hydrogel modified with nogo-66 receptor antibody and poly-L-lysine to promote axon regrowth after spinal cord injury. J Biomed Mater Res Part B: Appl Biomater 95, 110–117 (2010). 20725955 10.1002/jbm.b.31689

[b9] Guo J. . Reknitting the injured spinal cord by self-assembling peptide nanofiber scaffold. Nanomedicine: nanotechnology, biology, and medicine 3, 311–321, doi: 10.1016/j.nano.2007.09.003 (2007). 17964861

[b10] Cigognini D. . Evaluation of Early and Late Effects into the Acute Spinal Cord Injury of an Injectable Functionalized Self-Assembling Scaffold. PLoS ONE 6, e19782, doi: 10.1371/journal.pone.0019782 (2011). 21611127 PMC3097206

[b11] Hurtado A. . Robust CNS regeneration after complete spinal cord transection using aligned poly-L-lactic acid microfibers. Biomaterials 32, 6068–6079, doi: 10.1016/j.biomaterials.2011.05.006 (2011). 21636129 PMC4163047

[b12] Gao M. . Templated agarose scaffolds for the support of motor axon regeneration into sites of complete spinal cord transection. Biomaterials 34, 1529–1536, doi: 10.1016/j.biomaterials.2012.10.070 (2013). 23182350 PMC3518618

[b13] Thomas A. M. . Channel density and porosity of degradable bridging scaffolds on axon growth after spinal injury. Biomaterials 34, 2213–2220, doi: 10.1016/j.biomaterials.2012.12.002 (2013). 23290832 PMC3552139

[b14] Gros T., Sakamoto J. S., Blesch A., Havton L. A. & Tuszynski M. H. Regeneration of long-tract axons through sites of spinal cord injury using templated agarose scaffolds. Biomaterials 31, 6719–6729 (2010). 20619785 10.1016/j.biomaterials.2010.04.035

[b15] Yoshii S., Ito S., Shima M., Taniguchi A. & Akagi M. Functional restoration of rabbit spinal cord using collagen-filament scaffold. Journal of tissue engineering and regenerative medicine 3, 19–25, doi: 10.1002/term.130 (2009). 19012267

[b16] Chew S. Y., Wen J., Yim E. K. F. & Leong K. W. Sustained Release of Proteins from Electrospun Biodegradable Fibers. Biomacromolecules 6, 2017–2024, doi: 10.1021/bm0501149 (2005). 16004440

[b17] Sing Yian C., Todd C. H., Chwee Teck L. & Kam W. L. Mechanical properties of single electrospun drug-encapsulated nanofibres. Nanotechnology 17, 3880 (2006). 19079553 10.1088/0957-4484/17/15/045 PMC2597803

[b18] Chew S. Y., Mi R., Hoke A. & Leong K. W. Aligned Protein–Polymer Composite Fibers Enhance Nerve Regeneration: A Potential Tissue-Engineering Platform. Advanced functional materials 17, 1288–1296, doi: 10.1002/adfm.200600441 (2007). 18618021 PMC2447933

[b19] Liao I. C., Chew S. Y. & Leong K. W. Aligned core–shell nanofibers delivering bioactive proteins. Nanomedicine : nanotechnology, biology, and medicine 1, 465–471, doi: 10.2217/17435889.1.4.465 (2006). 17716148

[b20] Cao H., Jiang X., Chai C. & Chew S. Y. RNA interference by nanofiber-based siRNA delivery system. Journal of Controlled Release 144, 203–212 (2010). 20138939 10.1016/j.jconrel.2010.02.003

[b21] Handarmin . Nanofibrous scaffold with incorporated protein gradient for directing neurite outgrowth. Drug Delivery and Translational Research 1, 147–160, doi: 10.1007/s13346-011-0017-3 (2011). 25788113

[b22] Rujitanaroj P.-o., Wang Y.-C., Wang J. & Chew S. Y. Nanofiber-mediated controlled release of siRNA complexes for long term gene-silencing applications. Biomaterials 32, 5915–5923 (2011). 21596430 10.1016/j.biomaterials.2011.04.065

[b23] Liu T., Xu J., Chan B. P. & Chew S. Y. Sustained release of neurotrophin-3 and chondroitinase ABC from electrospun collagen nanofiber scaffold for spinal cord injury repair. Journal of Biomedical Materials Research Part A 100A, 236–242, doi: 10.1002/jbm.a.33271 (2012). 22042649

[b24] Jiang X. . Nanofiber topography and sustained biochemical signaling enhance human mesenchymal stem cell neural commitment. Acta Biomaterialia 8, 1290–1302 (2012). 22154861 10.1016/j.actbio.2011.11.019

[b25] Rujitanaroj P.-o. . Controlling fibrous capsule formation through long-term down-regulation of collagen type I (COL1A1) expression by nanofiber-mediated siRNA gene silencing. Acta Biomaterialia 9, 4513–4524 (2013). 23036951 10.1016/j.actbio.2012.09.029 PMC3523808

[b26] Low W. C., Rujitanaroj P. O., Wang F., Wang J. & Chew S. Y. Nanofiber-mediated release of retinoic acid and brain-derived neurotrophic factor for enhanced neuronal differentiation of neural progenitor cells. Drug Deliv Transl Res 5, 89–100, doi: 10.1007/s13346-013-0131-5 (2015). 25787735

[b27] Segura T., Chung P. H. & Shea L. D. DNA delivery from hyaluronic acid-collagen hydrogels via a substrate-mediated approach. Biomaterials 26, 1575–1584, doi: 10.1016/j.biomaterials.2004.05.007 (2005). 15522759 PMC2648403

[b28] Wallace D. G. & Rosenblatt J. Collagen gel systems for sustained delivery and tissue engineering. Advanced Drug Delivery Reviews 55, 1631–1649 (2003). 14623405 10.1016/j.addr.2003.08.004

[b29] Tabata Y., Miyao M., Ozeki M. & Ikada Y. Controlled release of vascular endothelial growth factor by use of collagen hydrogels. Journal of biomaterials science. Polymer edition 11, 915–930 (2000). 11211086 10.1163/156856200744101

[b30] McTigue D. M., Horner P. J., Stokes B. T. & Gage F. H. Neurotrophin-3 and brain-derived neurotrophic factor induce oligodendrocyte proliferation and myelination of regenerating axons in the contused adult rat spinal cord. The Journal of neuroscience the official journal of the Society for Neuroscience 18, 5354–5365 (1998). 9651218 10.1523/JNEUROSCI.18-14-05354.1998 PMC6793495

[b31] Bamber N. I. . Neurotrophins BDNF and NT-3 promote axonal re-entry into the distal host spinal cord through Schwann cell-seeded mini-channels. The European journal of neuroscience 13, 257–268 (2001). 11168530

[b32] Schnell L., Schneider R., Kolbeck R., Barde Y. A. & Schwab M. E. Neurotrophin-3 enhances sprouting of corticospinal tract during development and after adult spinal cord lesion. Nature 367, 170–173, doi: 10.1038/367170a0 (1994). 8114912

[b33] Milbreta U. . Three-Dimensional Nanofiber Hybrid Scaffold Directs and Enhances Axonal Regeneration after Spinal Cord Injury. ACS Biomaterials Science & Engineering 2, 1319–1329, doi: 10.1021/acsbiomaterials.6b00248 (2016). 33434985

[b34] Barres B. A. . A crucial role for neurotrophin-3 in oligodendrocyte development. Nature 367, 371–375, doi: 10.1038/367371a0 (1994). 8114937

[b35] Kumar S., Kahn M. A., Dinh L. & de Vellis J. NT-3-mediated TrkC receptor activation promotes proliferation and cell survival of rodent progenitor oligodendrocyte cells *in vitro* and *in vivo*. Journal of neuroscience research 54, 754–765 (1998). 9856859 10.1002/(SICI)1097-4547(19981215)54:6<754::AID-JNR3>3.0.CO;2-K

[b36] Iyer A. N., Bellon A. & Baudet M.-L. microRNAs in axon guidance. Frontiers in Cellular Neuroscience 8, 1–12 (2014). 24672429 10.3389/fncel.2014.00078 PMC3953822

[b37] Hancock M. L., Preitner N., Quan J. & Flanagan J. G. MicroRNA-132 Is Enriched in Developing Axons, Locally Regulates Rasa1 mRNA, and Promotes Axon Extension. The Journal of Neuroscience 34, 66–78 (2014). 24381269 10.1523/JNEUROSCI.3371-13.2014 PMC3866495

[b38] Sasaki Y., Gross C., Xing L., Goshima Y. & Bassell G. J. Identification of axon-enriched microRNAs localized to growth cones of cortical neurons. Develop Neurobiol 74, 397–406 (2014). 10.1002/dneu.22113 PMC4111797 23897634

[b39] Verma P. . Axonal Protein Synthesis and Degradation Are Necessary for Efficient Growth Cone Regeneration. The Journal of Neuroscience 25, 331–342 (2005). 15647476 10.1523/JNEUROSCI.3073-04.2005 PMC3687202

[b40] Strickland I. T. . Axotomy-Induced miR-21 Promotes Axon Growth in Adult Dorsal Root Ganglion Neurons. PLoS One 6, e23423 (2011). 21853131 10.1371/journal.pone.0023423 PMC3154476

[b41] Zhou S. . microRNA-222 Targeting PTEN Promotes Neurite Outgrowth from Adult Dorsal Root Ganglion Neurons following Sciatic Nerve Transection. PLoS One 7, e44768 (2012). 23028614 10.1371/journal.pone.0044768 PMC3441418

[b42] Wu D. & Murashov A. K. MicroRNA-431 regulates axon regeneration in mature sensory neurons by targeting the Wnt antagonist *Kremen1*. Frontiers in Molecular Neuroscience 6, Article 35 (2013). 10.3389/fnmol.2013.00035 PMC3807041 24167472

[b43] Liu N.-K., Wang X.-F., Lu Q.-B. & Xu X.-M. Altered microRNA expression following traumatic spinal cord injury. Experimental Neurology 219, 424–429 (2009). 19576215 10.1016/j.expneurol.2009.06.015 PMC2810508

[b44] Strickland E. R. . MicroRNA dysregulation following spinal cord contusion: Implications for neural plasticity and repair. Neuroscience 186, 146–160 (2011). 21513774 10.1016/j.neuroscience.2011.03.063 PMC3155824

[b45] Yunta M. . MicroRNA dysregulation in the spinal cord follwoing traumatic injury. PLoS One 7, e34534 (2012). 22511948 10.1371/journal.pone.0034534 PMC3325277

[b46] Bhalala O. G. . microRNA-21 regulates astrocytic response following spinal cord injury. J. Neuroscience 32, 17935–17947 (2012). 23238710 10.1523/JNEUROSCI.3860-12.2012 PMC3538038

[b47] Vo N. . A cAMP-response element binding protein-induced microRNA regulates neuronal morphogenesis. PNAS 102, 16426–16431 (2005). 16260724 10.1073/pnas.0508448102 PMC1283476

[b48] Dajas-Bailador F. . microRNA-9 regulates axon extension and branching by targeting Map1b in mouse cortical neurons. Nature Neuroscience 15, 697 (2012). 10.1038/nn.3082 22484572

[b49] Liu X. Q., Xiong M. H., Shu X. T., Tang R. Z. & Wang J. Therapeutic delivery of siRNA silencing HIF-1 alpha with micellar nanoparticles inhibits hypoxic tumor growth. Molecular pharmaceutics 9, 2863–2874, doi: 10.1021/mp300193f (2012). 22924580

[b50] Xiao C.-S., Wang Y.-C., Du J.-Z., Chen X.-S. & Wang J. Kinetics and Mechanism of 2-Ethoxy-2-oxo-1,3,2-dioxaphospholane Polymerization Initiated by Stannous Octoate. Macromolecules 39, 6825–6831, doi: 10.1021/ma0615396 (2006).

[b51] Jha B. S. . Two pole air gap electrospinning: Fabrication of highly aligned, three-dimensional scaffolds for nerve reconstruction. Acta Biomaterialia 7, 203–215 (2011). 20727992 10.1016/j.actbio.2010.08.004

[b52] Hou S. . The repair of brain lesion by implantation of hyaluronic acid hydrogels modified with laminin. Journal of Neuroscience Methods 148, 60–70 (2005). 15978668 10.1016/j.jneumeth.2005.04.016

[b53] Stitzel J. . Controlled fabrication of a biological vascular substitute. Biomaterials (2005). 10.1016/j.biomaterials.2005.07.048 16131465

[b54] Taylor, S. J., III, J. W. M. & Sakiyama-Elbert S. E. Controlled release of neurotrophin-3 from fibrin gels for spinal cord injury. Journal of Controlled Release 98, 281–294 (2004). 15262419 10.1016/j.jconrel.2004.05.003

[b55] Taylor S. J., Rosenzweig, E. S., III, J. W. M. & Sakiyama-Elbert S. E. Delivery of neurotrophin-3 from fibrin enhances neuronal fiber sprouting after spinal cord injury. Journal of Controlled Release 113, 226–235 (2006). 16797770 10.1016/j.jconrel.2006.05.005 PMC1615967

[b56] Johnson P. J., Tatara A., McCreedy D. A., Shiu A. & Sakiyama-Elbert S. E. Tissue-engineered fibrin scaffolds containing neural progenitors enhance functional recovery in a subacute model of SCI. Soft Matter 6, 5127–5137 (2010). 21072248 10.1039/c0sm00173b PMC2975358

[b57] Hejcˇl A. . HPMA-RGD Hydrogels Seeded with Mesenchymal Stem Cells Improve Functional Outcome in Chronic Spinal Cord Injury. Stem cells adn development 19, 1535 (2010). 10.1089/scd.2009.0378 20053128

[b58] Kubinova S. . Highly superporous cholesterol-modified poly(2-hydroxyethyl methacrylate) scaffolds for spinal cord injury repair. J Biomed Mater Res Part A 00, 00 (2011). 10.1002/jbm.a.33221 21953978

[b59] Guo J. . Reknitting the injured spinal cord by self-assembling peptide nanofiber scaffold. Nanomedicine: Nanotechnology, Biology, and Medicine 3, 311–321 (2007). 10.1016/j.nano.2007.09.003 17964861

[b60] Cigognini D. . Evaluation of Early and Late Effects into the Acute Spinal Cord Injury of an Injectable Functionalized Self-Assembling Scaffold. PLoS One 6, e19782 (2011). 21611127 10.1371/journal.pone.0019782 PMC3097206

[b61] Singh B. . Regeneration of diabetic axons is enhanced by selective knockdown of the PTEN gene. Brain 137, 1051–1067 (2014). 24578546 10.1093/brain/awu031 PMC3959560

[b62] Piantino J., Burdick J. A., Goldberg D., Langer R. & Benowitz L. I. An injectable, biodegradable hydrogel for trophic factor delivery enhances axonal rewiring and improves performance after spinal cord injury. Experimental Neurology 201, 359–367 (2006). 16764857 10.1016/j.expneurol.2006.04.020

[b63] Taylor S. J., Rosenzweig E. S., McDonald Iii J. W. & Sakiyama-Elbert S. E. Delivery of Neurotrophin-3 from Fibrin Enhances Neuronal Fiber Sprouting After Spinal Cord Injury. Journal of controlled release: official journal of the Controlled Release Society 113, 226–235, doi: 10.1016/j.jconrel.2006.05.005 (2006). 16797770 PMC1615967

[b64] Asanuma H. In Neural Mechanisms of Conditioning (eds Alkon DanielL & Woody CharlesD) Ch. 10, 187–196 (Springer: US, 1986).

[b65] Raineteau O., Fouad K., Noth P., Thallmair M. & Schwab M. E. Functional switch between motor tracts in the presence of the mAb IN-1 in the adult rat. Proceedings of the National Academy of Sciences of the United States of America 98, 6929–6934, doi: 10.1073/pnas.111165498 (2001). 11381120 PMC34455

[b66] Bareyre F. M. . The injured spinal cord spontaneously forms a new intraspinal circuit in adult rats. Nat Neurosci 7, 269–277 (2004). 14966523 10.1038/nn1195

[b67] van den Brand R. . Restoring Voluntary Control of Locomotion after Paralyzing Spinal Cord Injury. Science 336, 1182–1185 (2012). 22654062 10.1126/science.1217416

[b68] Tsuruta T. & Nakajima A. Multiphase Biomedical Materials. (Taylor & Francis, 1989).

[b69] Krebs M. D., Jeon O. & Alsberg E. Localized and sustained delivery of silencing RNA from macroscopic biopolymer hydrogels. Journal of the American Chemical Society 131, 9204–9206, doi: 10.1021/ja9037615 (2009). 19530653

[b70] Vo T. N., Kasper F. K. & Mikos A. G. Strategies for Controlled Delivery of Growth Factors and Cells for Bone Regeneration. Advanced drug delivery reviews 64, 1292–1309, doi: 10.1016/j.addr.2012.01.016 (2012). 22342771 PMC3358582

[b71] Richardson T. P., Peters M. C., Ennett A. B. & Mooney D. J. Polymeric system for dual growth factor delivery. Nature biotechnology 19, 1029–1034, doi: 10.1038/nbt1101-1029 (2001). 11689847

[b72] Chen F.-M., Zhang M. & Wu Z.-F. Toward delivery of multiple growth factors in tissue engineering. Biomaterials 31, 6279–6308 (2010). 20493521 10.1016/j.biomaterials.2010.04.053

[b73] Lee K., Silva E. A. & Mooney D. J. Growth factor delivery-based tissue engineering: general approaches and a review of recent developments. Journal of The Royal Society Interface 8, 153–170, doi: 10.1098/rsif.2010.0223 (2011). PMC3033020 20719768

[b74] Almad A., Sahinkaya F. R. & McTigue D. M. Oligodendrocyte Fate after Spinal Cord Injury. Neurotherapeutics 8, 262–273, doi: 10.1007/s13311-011-0033-5 (2011). 21404073 PMC3101831

[b75] Diao H. J., Low W. C., Lu Q. R. & Chew S. Y. Topographical effects on fiber-mediated microRNA delivery to control oligodendroglial precursor cells development. Biomaterials 70, 105–114 (2015). 26310106 10.1016/j.biomaterials.2015.08.029 PMC4769075

