# Author Correction: Three-dimensional aligned nanofibers-hydrogel scaffold for controlled non-viral drug/gene delivery to direct axon regeneration in spinal cord injury treatment

**DOI:** 10.1038/s41598-018-31314-8

**Published:** 2018-08-24

**Authors:** Lan Huong Nguyen, Mingyong Gao, Junquan Lin, Wutian Wu, Jun Wang, Sing Yian Chew

**Affiliations:** 1 School of Chemical and Biomedical Engineering, Nanyang Technological University, Singapore, 637459 Singapore; 2 Department of Spine Surgery, Renmin Hospital of Wuhan University, Wuhan, 430060 China; 3 School of Biomedical Sciences, The University of Hong Kong Li Ka Shing Faculty of Medicine, Pokfulam, Hong Kong SAR China; 4 Research Center of Reproduction, Development and Growth, Li Ka Shing Faculty of Medicine, The University of Hong Kong, Pokfulam, Hong Kong SAR China; 5 State Key Laboratory of Brain and Cognitive Sciences, Li Ka Shing Faculty of Medicine, The University of Hong Kong, Pokfulam, Hong Kong SAR China; 6 Guangdong-Hongkong-Macau Institute of CNS Regeneration, Jinan University, Guangzhou, 510632 PR China; 7 The CAS Key Laboratory of Innate Immunity and Chronic Disease, School of Life Sciences and Medical Center, University of Science & Technology of China, Hefei, Anhui 230027 PR China; 8 Lee Kong Chian School of Medicine, Nanyang Technological University, Singapore, 308232 Singapore; 9 Present Address: Department of spinal surgery, Renmin Hospital of Wuhan University, Wuhan, 430060 PR China

Correction to: *Scientific Reports* 10.1038/srep42212, published online 07 February 2017

This Article contains typographical errors. In the Introduction section,

“Although the direct administration of biological factors to injury sites is frequently applied, such an approach often does not lead to robust tissue regeneration and reformation due to the rapid biological clearance of these agents from our bodies^1,2,3,4^. Given this limitation, biodegradable scaffolds are increasingly employed as temporary frameworks for sustained delivery of biomolecules and to support neo-tissue formation.”

should read:

“Although the direct administration of biological factors to injury sites is frequently applied, such an approach often does not lead to robust tissue regeneration and reformation due to the degradation and rapid biological clearance of these agents from our bodies^1,2,3^. Given this limitation, biodegradable scaffolds are increasingly employed as temporary frameworks for sustained delivery of biomolecules and to support neo-tissue formation^4^.”

“On the other hand, miR-222 is enriched in axons and participates in controlling local protein synthesis at distal axons^36-38^. Such controlled local protein synthesis plays crticial roles in allowing severed axons to undergo regeneration within hours after injuries, independent of protein transport from the cell soma of neurons^36,39^. Unfortunately, the expression of miR-222 is often significantly altered after nerve injuries^40-46^. *In vitro*, when miR-222 was deliberately over-expressed in injured adult neurons, enhanced regrowth was observed^40,41,47,48^.”

should read:

“On the other hand, miR-222^41^ is one of the miRs that is enriched in axons and participates in controlling local protein synthesis at distal axons^36-38,41^. Such controlled local protein synthesis plays crticial roles in allowing severed axons to undergo regeneration within hours after injuries, independent of protein transport from the cell soma of neurons^36,39^. Unfortunately, the expression of these miRs^40–46^, and in particular, miR-222^41^ is often significantly altered after nerve injuries. *In vitro*, when miR-222 was deliberately over-expressed in injured adult neurons, enhanced regrowth was observed^41^.”

In the Materials and Methods section, under subheading ‘Aligned PCLEEP fibers’,

“The PCLEEP copolymer (Mw = 59,102, Mn = 25,542) was synthesized by previously reported methods^43,50^.”

should read:

“The PCLEEP copolymer (Mw = 59,102, Mn = 25,542) was synthesized by previously reported methods^50^.”

In the Results and Discussion section,

“Among the scaffolds analyzed, hydrogels^8,14,52,53,54,55,56,57,58^ and self-assembled peptide nanofibers^59,60^ are most popular.”

should read:

“Among the scaffolds analyzed, hydrogels^7,8,14,52,55,56,57,58^ and self-assembled peptide nanofibers^9,10^ are most popular.”

“Similarly, the fibrin scaffold by Taylor *et al*. could only deliver NT-3 for 2 weeks post-implantation due to the rapid scaffold degradation rate^63^.”

should read:

“Similarly, the fibrin scaffold by Taylor *et al*. could only deliver NT-3 for 2 weeks post-implantation due to the rapid scaffold degradation rate^55^.”

“Such controlled local protein synthesis at distal axons has allowed severed axons to undergo regeneration within hours after injuries^35,45^. This local protein synthesis is finely controlled by microRNAs, such as miR-222. Indeed, miR-222 enhanced axonal regrowth in injured adult neurons *in vitro*^40,41,47,48^.”

should read:

“Such controlled local protein synthesis at distal axons has allowed severed axons to undergo regeneration within hours after injuries^36,39^. This local protein synthesis is finely controlled by microRNAs^40,41,47,48^, such as miR-222^41^. Indeed, miR-222 enhanced axonal regrowth in injured adult neurons *in vitro*^41^.”

“To replenish this OL pool, oligodendrocyte precursor cells (OPCs) proliferate and are recruited to the lesion site for differentiation and remyelination^74,75^.”

should read:

“To replenish this OL pool, oligodendrocyte precursor cells (OPCs) proliferate and are recruited to the lesion site for differentiation and remyelination^74^.”

In the References section, reference 50 was incorrectly given as:

Xiao, C.-S., Wang, Y.-C., Du, J.-Z., Chen, X.-S. & Wang, J. Kinetics and Mechanism of 2-Ethoxy-2-oxo-1,3,2-dioxaphospholane Polymerization Initiated by Stannous Octoate. *Macromolecules* **39**, 6825–6831, doi: 10.1021/ma0615396 (2006).

The correct reference is listed below as reference 1.

In addition, references 54, 59, 60 and 63 are duplicates of references 7, 9, 10 and 55 respectively.
