# Supplementary material for: Three-dimensional aligned nanofibers-hydrogel scaffold for controlled non-viral drug/gene delivery to direct axon regeneration in spinal cord injury treatment
Source: Sci Rep. 2017 Feb 7;7:42212. doi: 10.1038/srep42212 (PMC5294639; doi:10.1038/srep42212)
Supplement: Supplementary Information [file srep42212-s1.pdf]

Supplementary Information

Three-dimensional aligned nanofibers-hydrogel scaffold for controlled non-viral drug/gene delivery to direct axon regeneration in spinal cord injury treatment

Lan Huong Nguyen [a, 1], *Mingyong Gao* [a, b, 1], *Junquan Lin* [a], *Wutian Wu* [c, d, e, f], *Jun Wang* [g], *Sing Yian Chew* [a, h, *]

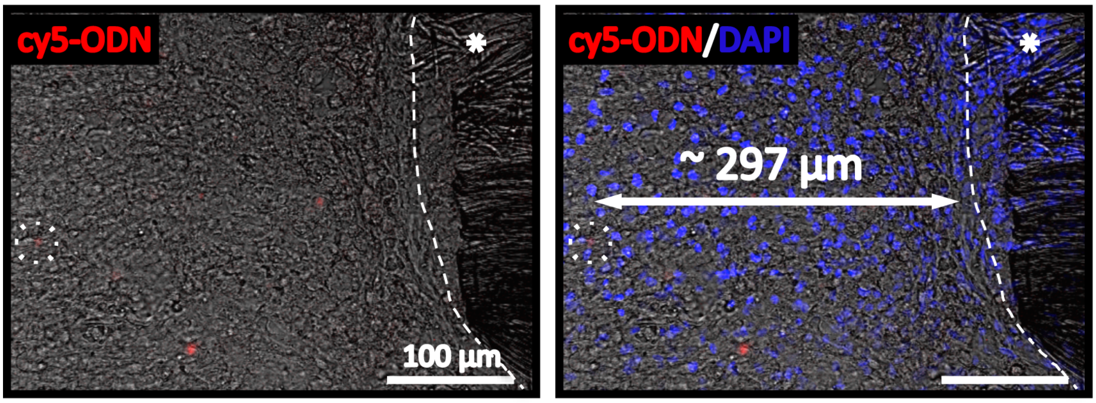

Supplementary Figure 1. microRNA distribution within spinal cord tissues after implanting Cy5-ODN incorporated nanofibers-hydrogel scaffolds for 7 days. Left: Cy5-ODN uptake (red, circled) in surrounding spinal cord tissues. Right: Merged image of Cy5-ODN and DAPI staining depicting the proximity between the released cy5-ODN and scaffold. Dotted line: tissue-implant interface. *: scaffold.
